# A New Optical Fiber Probe-Based Quantum Dots Immunofluorescence Biosensors in the Detection of *Staphylococcus aureus*

**DOI:** 10.3389/fcimb.2021.665241

**Published:** 2021-05-31

**Authors:** Jiewei Cui, Minjuan Zhou, Ying Li, Zhixin Liang, Yanqin Li, Ling Yu, Yang Liu, Yuan Liang, Liangan Chen, Changxi Yang

**Affiliations:** ^1^ Department of Pulmonary and Critical Care Medicine, Chinese PLA General Hospital, Beijing, China; ^2^ Laser Institute, Qilu University of Technology (Shandong Academy of Sciences), Qingdao, China; ^3^ State Key Laboratory of Precision Measurement Technology and Instrument, Department of Precision Instruments, Tsinghua University, Beijing, China; ^4^ Department of Respiratory and Critical Care Medicine, Beijing Chao-Yang Hospital, Capital Medical University, Beijing, China

**Keywords:** *Staphylococcus aureus*, biosensor, quantum dots, sensitivity, specificity

## Abstract

*Staphylococcus aureus* (*S. aureus*) is one of the most common clinical pathogenic bacteria with strong pathogenicity and usually leads to various suppurative infections with high fatality. Traditional bacterial culture for the detection of *S. aureus* is prone to diagnosis and antimicrobial treatment delays because of its long-time consumption and low sensitivity. In this study, we successfully developed a quantum dots immunofluorescence biosensor for *S. aureus* detection. The biosensor combined the advantages of biosensors with the high specificity of antigen-antibody immune interactions and the high sensitivity and stability of quantum dots fluorescence. The results demonstrated that the biosensor possessed high specificity and high sensitivity for *S. aureus* detection. The detection limit of *S. aureus* reached 1 × 10^4^ CFU/ml or even 1 × 10^3^ CFU/ml, and moreover, the fluorescence intensity had a significant positive linear correlation relationship with the logarithm of the *S. aureus* concentration in the range of 10^3^–10^7^ CFU/ml (correlation coefficient *R* ^2^ = 0.9731, *P* = 0.011). A specificity experiment showed that this biosensor could effectively distinguish *S. aureus* (1 × 10^4^ CFU/ml and above) from other common pathogenic (non-*S. aureus*) bacteria in nosocomial infections, such as *Klebsiella pneumoniae*, *Pseudomonas aeruginosa*, *Acinetobacter baumannii* and *Escherichia coli*. Additionally, the whole detection procedure spent only 2 h. In addition, the biosensor in this study may not be affected by the interference of the biofilm or other secretions since the clinical biological specimens are need to be fully liquefied to digest and dissolve viscous secretions such as biofilms before the detection procedure of the biosensor in this study. In conclusion, the biosensor could meet the need for rapid and accurate *S. aureus* detection for clinical application.

## Introduction

*Staphylococcus aureus* (*S. aureus*) is one of the most common pathogenic bacteria in nosocomial infections and accounted for 9.0% among all clinical isolates of strains in China reported by the Antimicrobial Surveillance Network (CHINET) in 2018 (Hu et al., 2018). Due to its production of various toxins and invasive enzymes, *S. aureus* usually leads to various suppurative infections, such as scabies, carbuncles, traumatic infections, pneumonia and sepsis, and even septic shock with high mortality (Gries et al., 2020; Karer et al., 2020). The traditional detection method for *S. aureus*, bacterial culture is prone to diagnosis and antimicrobial treatment delays because of its long-time consumption and low sensitivity (Stürenburg, 2009; Tenover et al., 2019). Therefore, rapid and accurate *S. aureus* detection is of great significance for anti-infection efforts to reduce its fatality.

Recently, many new detection methods, including molecular biology and immunological methods, have been applied to *S. aureus* detection (Tenover et al., 2019; Andersson et al., 2020). Among them, polymerase chain reaction (PCR) related molecular biology technology has high sensitivity and specificity (McIver et al., 2020), but its widespread clinical application is limited because a very small amount of contamination can easily lead to false positives (Renevey and Thur, 2018). However, immunofluorescence technology based on antigen-antibody specificity can ensure the high specificity required by the clinic (Nishiyama et al., 2020). Simultaneously, increasingly advanced fluorescence labeling technology and fluorescence detection technology have continuously increased the high sensitivity of immunofluorescence technology.

The development of fiber optic biosensor has received much attention. These biosensors with an optical fiber probe as the light carrier and the fluorescence detector have advantages to realize the compactness, miniaturization and portable of the instrument (Wolfbeis, 2000). They were widely applied in medical science (Ma et al., 2020), biological engineering (Yu et al., 2019), food industry (Chaudhari et al., 2020), and environmental monitoring (Zhou et al., 2021). We developed an optical fiber probe-based immunofluorescence biosensor that combined the advantages of antigen-antibody specificity, fluorescence labeling and transmission of fluorescence signals along the optical fiber. The surface of the probe of the biosensor was immobilized with *Escherichia coli*-specific antibodies, and the detection sensitivity for *E. coli* reached 10^3^ CFU/ml (CFU, colony forming units, an estimate of viable bacterial numbers usually existing in the form of colony), with high specificity (Liu et al., 2014; Zhang et al., 2014). However, the common fluorescence labelling techniques applied in their biosensor still have disadvantages, such as short fluorescence lifetime and spectral overlap affecting detection accuracy (Pu and Liu, 2009). In recent years, quantum dots have been increasingly used as fluorescence markers in labelling immunoassays (Song et al., 2017; Hao et al., 2020).

Quantum dots (QDs) is a new class of artificial luminescent fluorophores with the diameter of about 2–10 nm. QDs has obvious optical advantages that could facilitate the brighter emission and the more accurate detection of fluorescence signal, such as high fluorescence emission, long-life time with high photostability and large Stocks’ shifts (Wu et al., 2012; Abdelhamid and Wu, 2013). Additionally, the surface of QDs can be flexibly modified according to the needs of the application purpose and have been widely applied in various biological science researches. For example, Hani Nasser Abdelhamid and Hui-Fen Wu had reported a selective biosensing of *S. aureus* using QDs modified with Chitosan (Abdelhamid and Wu, 2017). Liu et al. (2011) had detected ferritin in human serum based on avidin–biotin system using the avidin-conjugated QDs. The results of these studies above had showed the optical advantages of quantum dots in biological detection.

Therefore, based on the abovementioned biosensor we developed and combined with the advantages of quantum dots fluorescence labeling technology, the purpose of this study is to achieve a fast, accurate sensing for *S. aureus* detection with high specificity and sensitivity.

## Methods

### Experimental Principle

**Figure 1:**
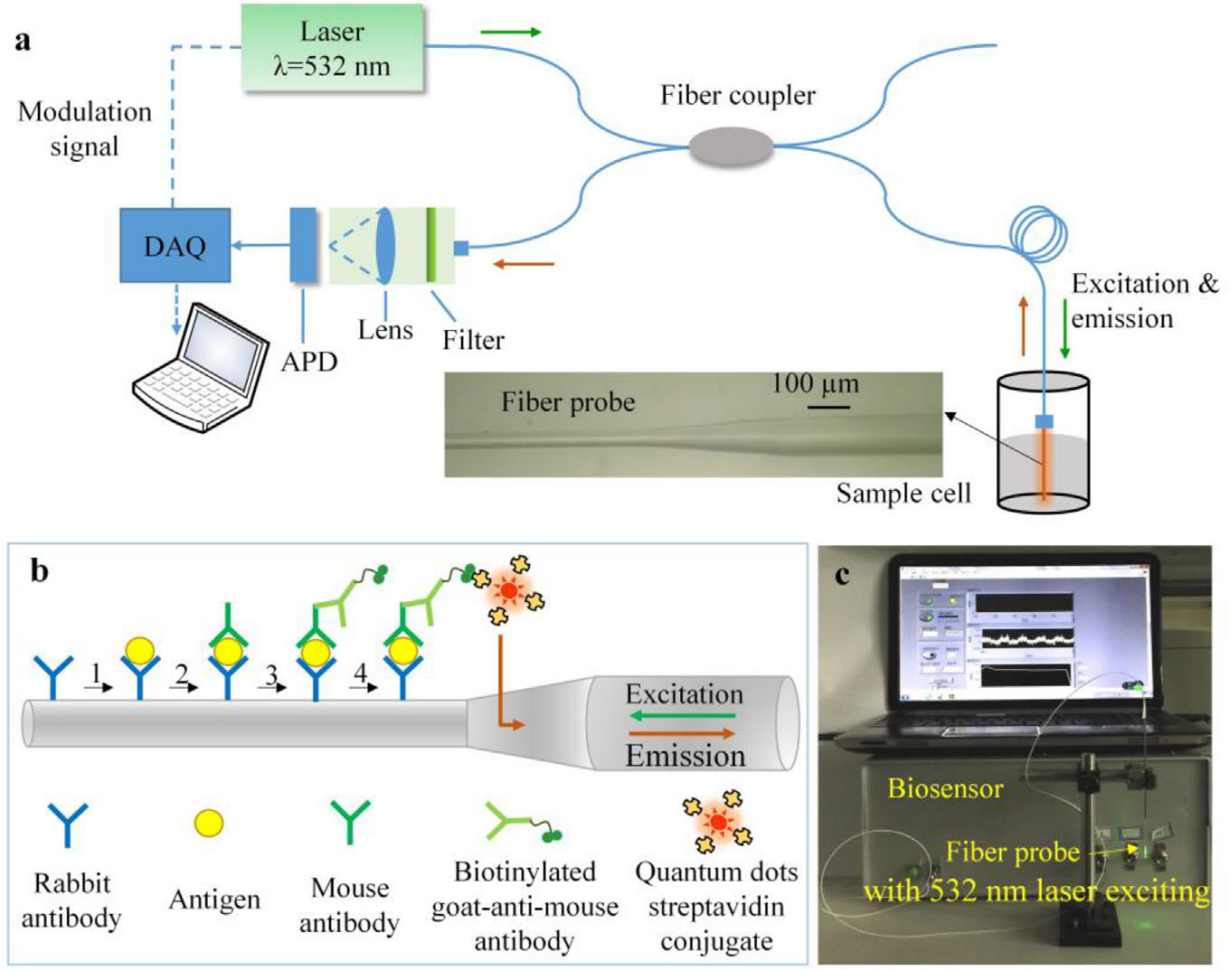
Principle of the fiber optic biosensor. **(A)** Schematic diagram of the fiber optic biosensor. A 532 nm laser was employed as excitation light that transmitted into the fiber probe through the fiber coupler. The fluorescence signal was collected and propagated back through the fiber coupler, filter, lens, avalanche photo-diode (APD), data acquisition (DAQ) system, and at last displayed on the computer. **(B)** The principle to identify *S. aureus* using the fiber probe. The rabbit polyclonal anti-*S. aureus* antibody immobilized on the probe surface was used to capture as many *S. aureus* as possible in order to improve the sensitivity. When the *S. aureus* strains were captured, the mouse monoclonal anti-*S. aureus* antibody specifically binds to the captured *S. aureus* again to form a double-antibody sandwich structure. The choice on the two antibodies could ensure that the probe has both high sensitivity and high specificity in capturing and detecting *S. aureus*. The biotin labeled goat-anti-mouse IgG antibody was bound to detecting antibody, and then avidin-labeled quantum dots (QDs) bound with the biotin and emitted fluorescence signal with the 532 nm laser excitation, the emitted fluorescence signal was collected at 605 nm wavelength. Moreover, the signal cascade amplification of the biotin–avidin–QDs system was used to enhance the fluorescence signal and improve the sensitivity. **(C)** Prototype of the fiber optic biosensor. All the components were encapsulated in the box except the fiber probe and sample cell. The fiber probe was fixed by a fluorescence connector to the fiber coupler by the fiber adapter in the front-panel of the box.

The structural schematic diagram of the fiber optic biosensor is shown in **Figure 1A**. A semiconductor laser (Shanghai Fiblaser Technology Co., Ltd., Shanghai, China) with wavelength of 532 nm and a modulation frequency of 1 kHz was selected as the excitation light source, because the excitation wavelength of QDs is ≤565 nm. The laser was linked to one input of an optical fiber coupler (2 × 2, couple ratio: 20:80) through its pigtail fiber. The combination tapered fiber probe was connected to the 20% end of the coupler using the fluorescence optical fiber connector. The input laser light was transmitted to the fiber probe to excite QDs that bound with antibody specifically captured on the fiber probe surface (**Figure 1B**). The fluorescence signal with 605 nm emission wavelength of QDs was also collected by the fiber probe, and sent back to the other input end of the fiber coupler. The fluorescence signal was filtered through a long-pass filter (FEL0600, Thorlabs, USA) with a cut-on wavelength of 600 nm in order to remove the excitation light (532 nm). Then, the fluorescence signal was injected into an avalanche photo-diode (APD, LCSA500-01, Laser Components, Germany) for photo-electronic conversion, and detection in data acquisition (DAQ) system. To enhance signal resolution, digital signals from DAQ system were further processed by a correlation detection system based on phase-locked filtering algorithm, which was performed with LabVIEW (Liu et al., 2014). Finally, the fluorescence signal was displayed on the computer screen. The fiber optic immunofluorescence biosensor prototype was shown in **Figure 1C**.

In this study, the immune sandwich principle of antibody–antigen specific capture of *S. aureus* was used to improve the sensitivity and specificity of *S. aureus* detection. We had selected two different kind of antibodies, among them, the rabbit polyclonal anti-*S. aureus* antibody (ab20920) immobilized on the probe surface was used to capture as many *S. aureus* as possible in order to improve the sensitivity of the biosensor. When the *S. aureus* strains were captured, the mouse monoclonal anti-*S. aureus* antibody (ab37644) specifically binds to the captured *S. aureus* again to form a double-antibody sandwich structure. Therefore, the choice on the two antibodies could ensure that the probe has both high sensitivity and high specificity in capturing and detecting *S. aureus*. As shown in **Figure 1B**, the activated fiber probe immobilized with rabbit polyclonal antibody to *S. aureus* (ab20920) defined as capture antibody was firstly used to capture *S. aureus*. Secondly, the fiber probe bound with bacteria was incubated in the mouse monoclonal antibody (ab37644), which was specific for *S. aureus* and defined as detecting antibody to bind to *S. aureus*. Thirdly, the biotin labelled goat-anti-mouse IgG antibody (GaMIgG) was introduced to bind to the mouse monoclonal antibody (ab37644). Finally, avidin-labelled QDs (605 nm emission wavelength) was employed for fluorescence labelling by the specific binding between biotin and avidin. Moreover, the signal cascade amplification principle of the biotin–avidin–QDs system was used to enhance the fluorescence signal and improve the sensitivity and stability of *S. aureus* detection.

### Experimental Materials

The fiber optic immunofluorescence biosensor was developed by the Department of Precision Instruments, Tsinghua University. The fiber probe was made of a step-index multimode silicon optical fiber (NA = 0.22, 105 μm core/125 μm cladding, Beijing Scitlion Technology Co., Ltd., Beijing, China), and the composition of the silicon is mainly Silicon dioxide (SiO2). The rabbit polyclonal anti-*S. aureus* antibody (ab20920) and mouse monoclonal anti-*S. aureus* antibody (ab37644) was purchased from Abcam (USA). Biotin labeled goat-anti-mouse IgG antibody (GaMIgG) and avidin-labeled QDs (QDs-605, its emission wavelength is 605 nm and its excitation wavelength of the laser in this study is 532 nm) were purchased from Wuhan Jiayuan Quantum Dots Co., Ltd., (Wuhan, China). The QDs labeled to avidin applied in this study is a kind of silicon (Si) nano quantum dot. Sodium borohydride (NaBH_4_) was purchased from Shanghai McLean Biochemical Technology Company, and Trypticase Soy Broth bacterial culture medium (TSB) was purchased from Beijing Aokexing Biotechnology Company. Bovine serum albumin (BSA, P1621), phosphate buffer (1×PBS, B1203), Phosphate Tween buffer (1×PBST, A1007), and other non-special reagents were purchased from Beijing Pulilai Gene Technology Corporation in China.

All experimental bacterial strains were derived from the clinical microbiological laboratory of our hospital (Chinese PLA General Hospital), including the *S. aureus* standard strain (strain number ATCC 29213), *Pseudomonas aeruginosa* (ATCC 27853), *Acinetobacter baumannii* (ATCC 19606), *E. coli* (ATCC 25922), *Staphylococcus epidermidis* (clinically isolated from a patient), *Klebsiella pneumoniae* (clinically isolated from a patient) and five clinical *S. aureus* strains isolated from sputum specimen of patients with chronic obstructive pulmonary disease complicated with pulmonary infection, the five clinical *S. aureus* strains were respectively named as SA-1, SA-2, SA-3, SA-4 and SA-5. Among the five clinical *S. aureus* strains, the two *S. aureus* strains (SA-4 and SA-5) were from patients with severe lung infection, and both the two *S. aureus* strains were sensitive to vancomycin according to the antimicrobial susceptibility results. The two patients were also treated with vancomycin for anti-infective treatment, but the clinical effect was evaluated to be ineffective after excluding the influence of other clinical factors. It was speculated that the two *S. aureus* strains might have produced the biofilm in the infection in body, resulting in ineffective antimicrobial treatment.

### Bacterial Preparation

Bacterial culture was performed in a biosafety cabinet in the clinical microbiological laboratory. The stored *S. aureus* standard strain (ATCC 29213 strain) was cultured on solid medium for 24 h by streaking to grow single colonies. A single colony was picked and inoculated in 20 ml of TSB liquid medium for 16 h to the logarithmic growth phase of the bacteria. The bacteria in the logarithmic growth phase were centrifuged at 5,000 revolutions per minute (rpm) for 5 min, and the supernatant was aspirated. The remaining bacteria were washed twice with PBS, and the bacterial concentration was determined using a haemocytometer. This method is to take a certain volume of cell suspension of the sample and place it in the counting chamber of a hemocytometer, and then observe and count by microscope. Since the volume of the counting chamber is constant, the number and the concentration of bacteria in the sample can be calculated according to the number of bacteria in the counting chamber of the hemocytometer. This method is simple and easy to implement, and the result can be obtained immediately. This method is not only suitable for cell and bacterial counting, but also for fungi and fungal spores. The concentration of the bacterial suspension was adjusted to 1 × 10^8^ CFU/ml with PBS and then serially diluted 10-fold six times for subsequent experiments, producing 10^8^, 10^7^, 10^6^, 10^5^, 10^4^, and 10^3^ CFU/ml concentrations.

Clinical isolates of the five *S. aureus* strains mentioned above were derived from sputum specimen of chronic obstructive pulmonary disease (AECOPD) patients by bacterial culture, and identified by automatic microorganism identification instrument (VITEK 2). Subsequently, the clinical isolated strains and other strains used for this study were also cultured in the same way as the *S. aureus* standard strain (ATCC 29213).

### Preparation of the Antibody-Immobilized Optical Fiber Probes of the Biosensor

A taper-and-cylinder combination optical fiber was prepared as the fiber probe. It was fabricated with a simple static tube etching method with 40% hydrofluoric acid (HF) as the etchant (Liu et al., 2014; Zhang et al., 2014). The fiber probe (inset in **Figure 1A**) consisted of a tapered section and a sensing region, whose length were approximately 0.03 and 1 cm, respectively. The diameter of tapered section was etched from 125 μm to about 45 μm within the length of 300 μm, and the diameter of sensing region was maintained about 45 μm for matching the V number of fiber (Anderson et al., 1994).

As shown in **Supplemental Material Figure S1**, the fiber probe was immersed in a piranha solution to remove the residual HF. And then, it was activated by hydroxylation, silanization, glutaraldehyde cross-linking, and covalent conjugation of the rabbit polyclonal anti-*S. aureus* antibody (ab20920, 20 × 10^−3^ g/ml in Tris–HCl buffer, which is the optimal concentration proved in the followed exploratory research) to capture the *S. aureus* specifically. After antibody immobilization, the fiber probe was immersed in NaBH_4_ solution to reduce the interference of background fluorescence caused by aldehyde group (Clancy and Cauller, 1998). And the remaining aldehyde groups and non-specific binding cites of the fiber probe were blocked by BSA solution. The activated fiber probes were stored at 4°C for future use.

### The Exploratory Research on the Optimal Concentration of the Antibody Used to Immobilize Onto the Probe Surface

As the same as the rabbit polyclonal anti-*S. aureus* antibody (ab20920) immobilized on the probe surface, the biotin-labeled goat anti-mouse IgG antibody used in this study was also a polyclonal antibody and it was labeled with biotin. Therefore, it was used as an analog to explore the optimal concentration of the rabbit polyclonal antibody (ab20920) used to immobilize onto the probe surface. The schematic diagram of experimental principle and the results were exhibited in **Supplemental Material Figure S2**. The processes of the research were as followed.

The biotin-labeled goat anti-mouse IgG antibody was diluted with Tris–HCl buffer to different antibody concentrations (2, 5, 10, 20, 50, 100 × 10^−3^ g/ml) as shown in the **Supplemental Material Figure S2** and the Tris–HCl buffer was as the control group without antibody. Firstly, the probe was incubated in the biotin-labeled goat anti-mouse IgG antibody in various concentrations mention in the **Supplemental Material Figure S2b** at 37°C overnight to allow the aldehyde groups on the probe to covalently bind amino groups of the antibody and made the antibody immobilized onto the probe surface. Secondly, after antibody immobilization, the fiber probe was immersed in NaBH4 solution to reduce the interference of background fluorescence caused by aldehyde group. And the remaining aldehyde groups and non-specific binding cites of the fiber probe were blocked by BSA solution. Thirdly, the probes were immersed in a biotin-labeled goat anti-mouse IgG antibody dilution solution (1:100 dilution) for 30 min at 37°C. Fourthly, the probes were washed with PBST and were immersed in the avidin-labeled quantum dots solution (QDs-605, 1:100 dilution) for 30 min at 37°C. Finally, the probes were washed with PBST for three times and then were connected to the biosensor and turn on the laser. After running 200 s, the fluorescence signal was collected and displayed on the computer. As proven in the exploratory experiment of the fluorescence signal-time response trace mentioned below, the effective response interval was defined from 50 s to 200 s and the average values of the fluorescence signal of the effective time interval calculated by the computer could be the optimal detection values. Each of the above experimental groups was repeated three times and their optimal detection values of the fluorescence signal were collected for statistical analysis of the differences between each group.

As shown in **Supplemental Material Figure S2**, the results had confirmed that the 20 × 10^−3^ g/ml was the optimal concentration of the antibody used to immobilize onto the probe surface.

### The SEM and EDX Images of the *S. aureus* Captured on the Optical Fiber Probes

The SEM images of the *S. aureus* captured on the optical fiber probes were performed to evaluate the capture capacity of the prepared antibody-immobilized optical fiber probes for *S. aureus*. There were six groups in the experiment. Among them, the five experimental groups (five groups) were the prepared *S. aureus* standard strain (ATCC 29213) with a concentration gradient of 10^8^–10^4^ CFU/ml. In addition, the control group (one group) was the undiluted *S. aureus* suspension (1 × 10^8^ CFU/ml) and the non-antibody-immobilized optical fiber probe, which was used to detect whether the non-antibody-immobilized optical fiber probe could capture non-*S. aureus* strains. Each probe was immersed in the 1 ml *S. aureus* suspension of the various concentrations for 30 min at 37°C, and then the probe was removed and washed three times with 1 ml of PBST to remove non-specific adsorption. All the probes were imaged by the scanning electron microscope (SEM, Hitachi S-5500). In addition, during the process of the SEM imaging of the probe surface, we also analyzed the energy spectrums of the SEM image of *S. aureus* captured on the surface of the fiber probe. Energy spectrums were recorded by the Energy dispersive X-ray spectrometry (EDX) combined with SEM (Hitachi S-5500). The principle of the EDX is as follow.

**Figure 2:**
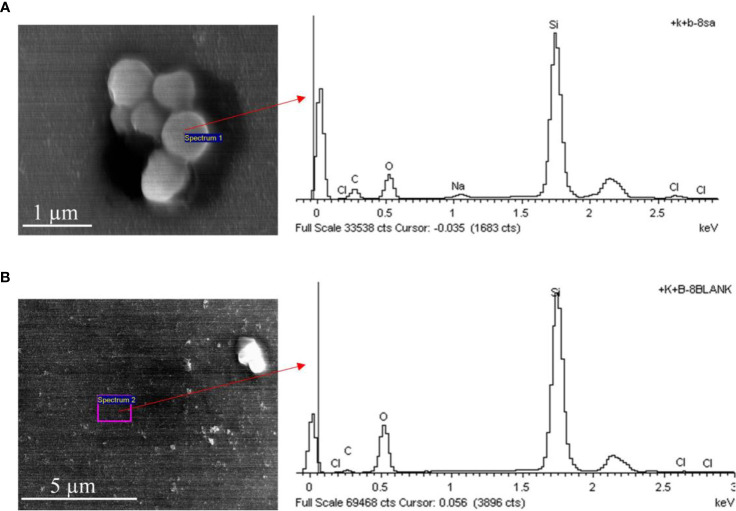
The SEM and EDX images of *S. aureus* and blank area on the surface of the fiber probe. **(A)** *S. aureus* captured on the fiber probe on the surface of the fiber probe. **(B)** The blank area of the fiber probe without *S. aureus*. The composition of the silicon optical fiber probe is mainly Silicon dioxide (SiO2), and the surface of the probe is rich in Si atoms and O atoms. While the *S. aureus* and other bacteria are organisms with carbohydrate as the main component, which is rich in Carbon (C) atoms and Sodium (Na) atoms. Therefore, the EDX image showed that the peak of the relative content (atomic percentage, Atomic %) of the C atoms and Na atoms were higher in the area where *S. aureus* were captured **(A)** than that in the blank area **(B)**. However, since the background of the SME imaging is the silicon optical fiber probe, the percentages of Si atoms and O atoms are still dominant, regardless of the presence or absence of *S. aureus* and other bacteria. By this way, the presence or absence of *S. aureus* and other bacteria on the surface of the probe can be distinguished. SEM, scanning electron microscope; EDX, energy dispersive x-ray spectrometry.

The research purpose of the SEM and EDX images is to evaluate whether there were captured bacteria and to exclude some non-bacterial false light spots in the SEM images using EDX. In all SEM images of various groups, there may be areas where bacteria are captured and blank areas where bacteria are not captured. The result exhibited in **Figure 2** was just used to prove that the EDX technology during SEM image analysis can ensure that SEM image analysis can effectively identify bacteria captured on the probe.

### The Detection Process of the Sample by the Biosensor and the Fluorescence Signal-Time Response Trace

Using the *S. aureus* standard strains, we exploratory studied the fluorescence signal responses corresponding to bacterial concentrations and the fluorescence signal-time response trace of the biosensor in the detection process. The exploratory experiment only included two samples. One sample was the *S. aureus* standard strains at the 1 × 10^7^ CFU/ml concentration, the other sample was PBS control without any bacteria (the concentration of the *S. aureus* was 0). The detection process of the samples was as follows.

Firstly, two prepared antibody-immobilized optical fiber probes were immersed in the two samples solutions above for 30 min at 37°C for *S. aureus* capture. Secondly, both the probes were washed three times with PBST to remove non-specific adsorption, and then the probes were immersed in the mouse monoclonal anti-*S. aureus* antibody dilution solution (ab37644, 100 × 10^−3^ g/ml) for 30 min at 37°C for formatting the double antibody sandwich structure. Thirdly, after washed with PBST, the probes were immersed in a biotin-labeled goat anti-mouse IgG antibody dilution solution (1:100 dilution) for 30 min at 37°C. Fourthly, after washed with PBST, the probes were immersed in an avidin-labeled quantum dots solution (QDs-605, 1:100 dilution) for 30 min at 37°C. Finally, the probes were washed with PBST and were connected to the biosensor. Then, turn on the laser with the 532 nm wavelength excitation light. The emitted fluorescence signal was collected at 605 nm wavelength for 200 seconds (200 s) or longer and the fluorescence signal-time response trace was displayed on the computer.

According to the signal-time response trace, the effective response interval could be defined from 50 to 200 s and the average values of the fluorescence signal of the effective time interval calculated by the computer could be the optimal detection values applied in this study.

### Biosensor Sensitivity Experiment

The sensitivity experiment was performed to evaluate the detection limit of *S. aureus* provided by the optical fiber probe-based fluorescence biosensor. There were seven groups in the experiment: the control group was PBS, and the other six experimental groups were the prepared *S. aureus* suspensions with the concentration gradients of 10^8^–10^3^ CFU/ml. The detection process of all the samples by the biosensor was as the same as the detection process in exploratory experiment of the fluorescence signal-time response trace mentioned above. And each of the above experimental groups was repeated three times and their optimal detection values of the fluorescence signal were collected for statistical analysis of the differences between each group.

### Biosensor Specificity Experiment

To evaluate the specificity of the biosensor, five most common clinical pathogenic non-*S. aureus* strains were employed in the experiment. The bacterial strains were *S. epidermidis*, *K. pneumoniae*, *P. aeruginosa*, *A. baumannii* and *E. coli.* All the strains were cultured to the logarithmic growth phase, and the bacterial suspension concentrations were all adjusted to 1 × 10^7^ CFU/ml with PBS. The experiment consisted of eight groups, including the five experimental groups (the five non-*S. aureus* strains mentioned above), two positive control groups (1 × 10^4^ CFU/ml and 1 × 10^7^ CFU/ml *S. aureus* suspensions) and 1 blank control group (PBS). The detection process of all the samples by the biosensor was as the same as the detection process in exploratory experiment of the fluorescence signal-time response trace mentioned above. And each of the above experimental groups was repeated three times and their optimal detection values of the fluorescence signal were collected for statistical analysis of the differences between each group.

### Clinical Application of the Biosensor

To verify the sensitivity (detection limit) of the biosensor for clinically isolated *S. aureus* strains, five *S. aureus* strains (named SA-1 to SA-5) isolated from different patients in our hospital were detected by the biosensor. Each of the 5 *S. aureus* strain suspensions above was adjusted to two concentrations (1 × 10^3^ CFU/ml and 1 × 10^4^ CFU/ml). A *S. aureus*-free PBS solution was used as a negative control group. Thus, a total of 11 groups were in the experiment. The detection process of all the samples by the biosensor was as the same as the detection process in exploratory experiment of the fluorescence signal-time response trace mentioned above. And each of the above experimental groups was repeated three times and their optimal detection values of the fluorescence signal were collected for statistical analysis of the differences between each group.

### Statistical Analysis

SPSS 19.0 software (IBM Corp., Armonk, NY, USA) was used for statistical analysis of the experimental data in this study. All data are tested for normal distribution and homogeneity of variance. All data conforming to the normal distribution were analyzed by two independent sample t-tests for statistical differences between groups. On the contrary, the rank sum test is used to analyze the statistical differences between groups for non-normally distributed data. For the linear regression relationship between the fluorescence intensity and the concentration gradient of *S. aureus*, the linear regression equation was established and the correlation coefficient (r) was calculated. All statistical analyses were performed using two-sided tests, and *P <*0.05 was considered to be statistically significant.

## Results

### The SEM and EDX Images of *S. aureus* Captured by the Antibody-Immobilized Optical Fiber Probes

**Figure 3:**
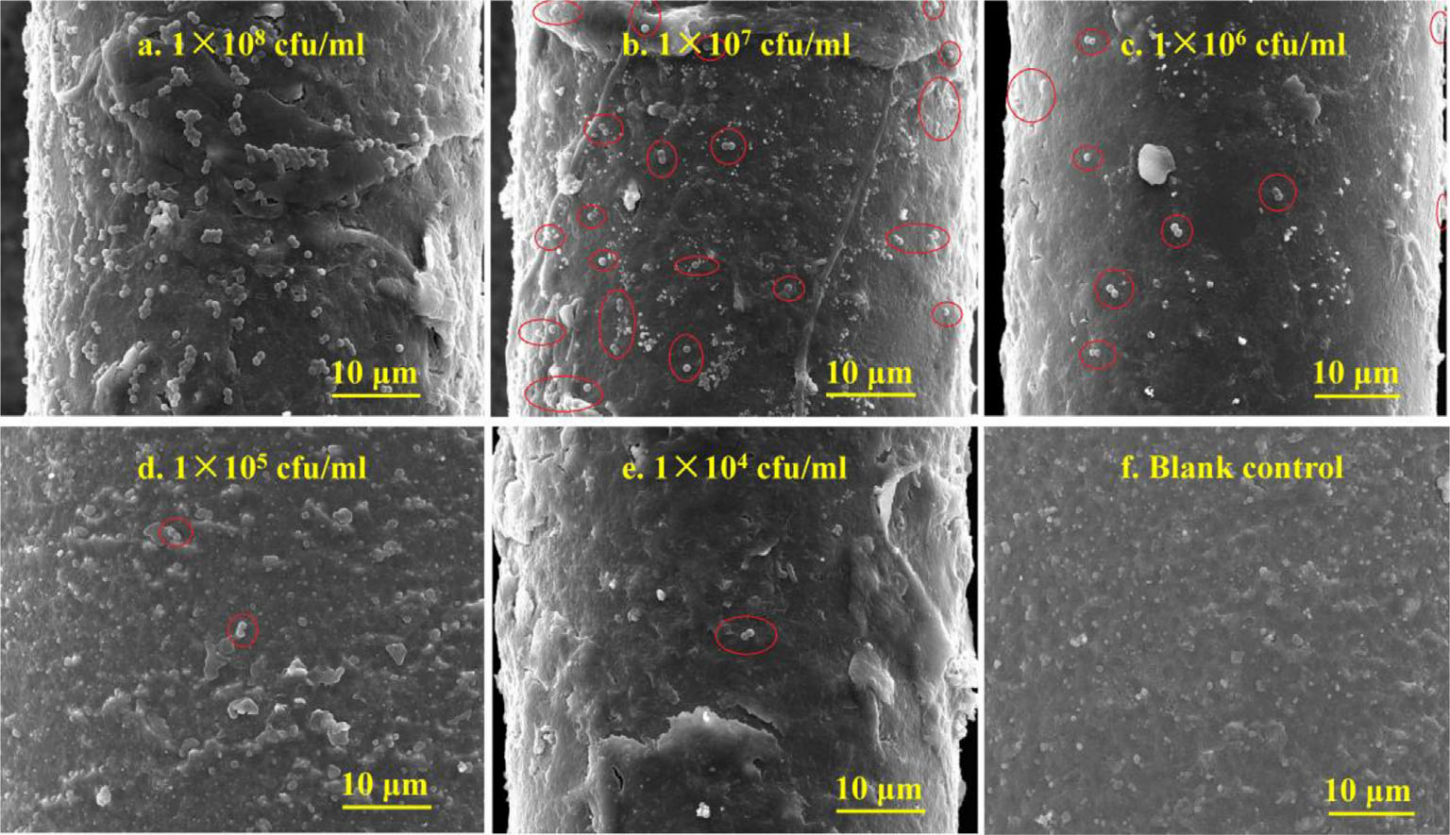
The SEM images of the *S. aureus* captured by the antibody-immobilized optical fiber probes. Figures **(A–E)** show the captured images of bacterial concentration from 1 × 10^8^ to 1 × 10^4^ CFU/ml respectively, while Figure **(F)** shows the blank control group without bacterial capture. The probes could capture more *S. aureus* with the increase of the concentration gradients of the *S. aureus* suspension.

As exhibited in **Figure 3**, the SEM images of the optical fiber probes capturing *S. aureus* show that the higher the concentration of the *S. aureus* suspension is, the more *S. aureus* can be captured by the probes. However, with the increase of the *S. aureus* suspension concentration (from 1 × 10^4^ to 1 × 10^8^ CFU/ml), the capture amounts of the *S. aureus* strain on the surface of the probes gradually increased in *S. aureus* suspensions at various concentrations.

In addition, the energy spectrums were recorded by the energy dispersive X-ray spectrometry (EDX) combined with SEM images. The comparation of the SEM and EDX image of between *S. aureus* and the blank area on the surface of the fiber probe were shown in **Figure 2**. The precise EDX analysis is shown in **Supplemental Material Table S1**. And the results suggested that the *S. aureus* strain contain Carbon (C) atoms and Sodium (Na) atoms, and the mass percentage (Weight %) and atomic percentage (Atomic %) of carbon (C) and sodium (Na) were higher than the blank area of the fiber probe. By this way, the presence or absence of *S. aureus* and other bacteria on the surface of the probe can be distinguished.

### The Fluorescence Signal-Time Response Trace and the Optimal Detection Values of the Biosensor

**Figure 4:**
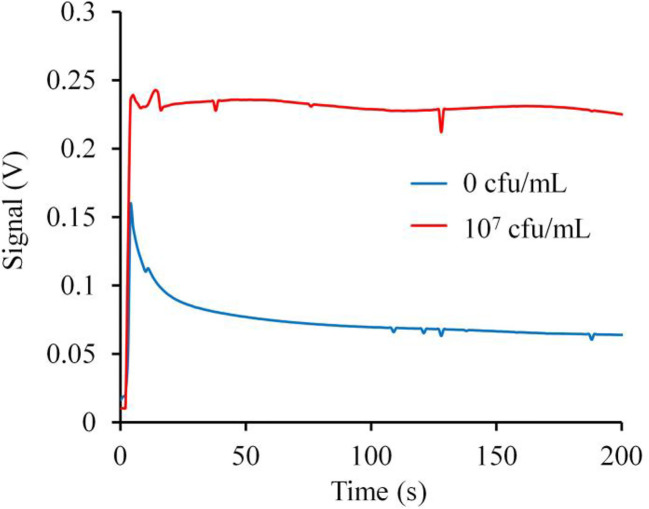
The fluorescence signal-time response trace and the optimal detection values applied in this study. The time response curve shows that the signal reached the maximum value instantly after the laser turning on and then decreased back gradually. After decreasing back, the fluorescence signal gradually tended to be stable. The trace showed that the effective response interval could be defined from 50 to 200 s and the average values of the fluorescence signal of the effective time interval calculated by the computer could be the optimal detection values applied in this study. In addition, the results also showed that the signal response of the *S. aureus* at the 10^7^ CFU/ml concentration was significantly higher than that of 0 CFU/mL and the biosensor might be used to distinguish *S. aureus* at different concentrations.

The fluorescence signal-time response trace during a complete test cycle (200 s) was shown in **Figure 4**. The time response curve shows that the signal reached the maximum value instantly after the laser turning on and then decreased back gradually. This was caused by photo-bleaching (Liu et al., 2014; Zhang et al., 2014). After decreasing back, the fluorescence signal gradually tended to be stable. The burr phenomenon on the curve may be the electrical signal interference during the instrument operation. The results of the trace showed that the effective response interval could be defined from 50 to 200 s and the average values of the fluorescence signal of the effective time interval calculated by the computer could be the optimal detection values applied in this study. In addition, the results also showed that the signal response of the *S. aureus* at the 10^7^ CFU/ml concentration was significantly higher than that of 0 CFU/ml (PBS control without any bacteria) and the biosensor might be used to distinguish *S. aureus* at different concentrations.

### Biosensor Sensitivity Results

**Figure 5:**
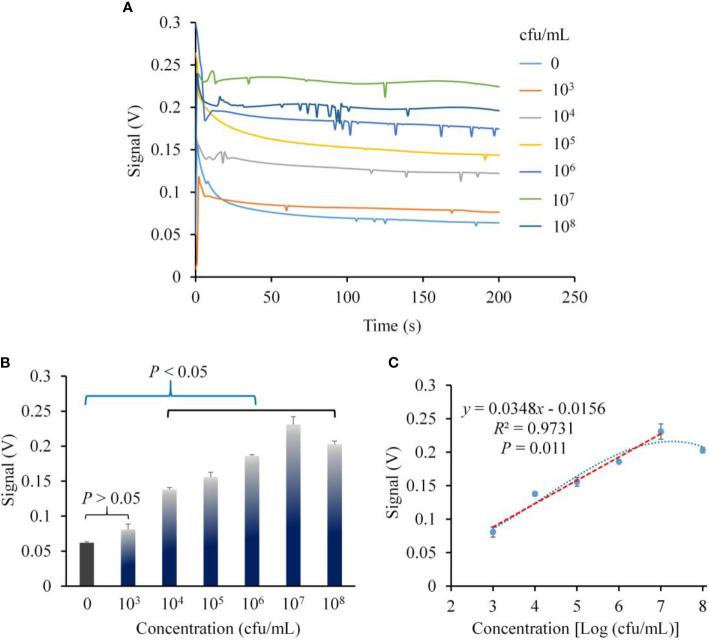
The sensitivity of the biosensor for the detection of *S. aureus*. **(A)** Signal-time response trace during a complete test cycle (200 seconds, 200 s) with the bacterial concentration gradients from 0 to 10^8^ CFU/ml, and the signals value at the 200 s were applied to the statistical analysis in **(B)** and **(C, B)** The optimal signal detection values of the fluorescence intensity of *S. aureus* at various concentration gradients and their statistical analysis showed that the sensitivity was 10^4^ CFU/ml. **(C)** The linear regression curve of signal versus the logarithm of the *S. aureus* concentration gradients. There was a significant positive linear correlation relationship with the fluorescence intensity in the range of 10^3^–10^7^ CFU/ml concentration (correlation coefficient *R* ^2^ = 0.9731, *P* = 0.011). Error bars in **(B, C)** are based on standard deviations (n = 3).

The signal corresponding to fluorescence intensity and significant difference between the *S. aureus* concentration groups are shown in **Figures 5A, B**. Signal-time response trace during a complete test cycle (200 s) with the *S. aureus* concentration gradients from 0 to 1 × 10^8^ CFU/ml is shown in **Figure 5A**. The histogram in **Figure 5B** shows the average signal measured in triplicate at different concentrations of *S. aureus*. Compared with the signal of the blank control group, the fluorescence intensity of the 1 × 10^3^ CFU/ml concentration group was higher but not significantly (*P >*0.05). However, the fluorescence intensity of all concentrations ≥1 × 10^4^ CFU/ml was significantly higher than that of the blank control (*P <*0.05). Moreover, in the concentration range of 1 × 10^4^ CFU/ml to 1 × 10^7^ CFU/ml, the fluorescence intensity gradually increased with increasing *S. aureus* concentration, and there was a dose-effect relationship. Surprisingly, the fluorescence intensity of the 1 × 10^8^ CFU/ml group was lower than that of the 1 × 10^7^ CFU/ml experimental group.

To evaluate the linear correlation between fluorescence intensity and *S. aureus* concentration, a linear regression curve of fluorescence intensity and *S. aureus* concentrations was established (**Figure 5C**). The results showed that the logarithm of the *S. aureus* concentration had a significant positive linear correlation relationship with the fluorescence intensity in the range of 10^3^–10^7^ CFU/ml (correlation coefficient *R* ^2^ = 0.9731, *P* = 0.011). The linear regression equation is *y* = 0.0348*x* − 0.0156. Therefore, this biosensor could achieve quantitative detection of *S. aureus*. Correspondingly, the approximate concentration of *S. aureus* in the samples to be tested might be estimated by the fluorescence intensity.

### Biosensor Specificity Experiment

**Figure 6:**
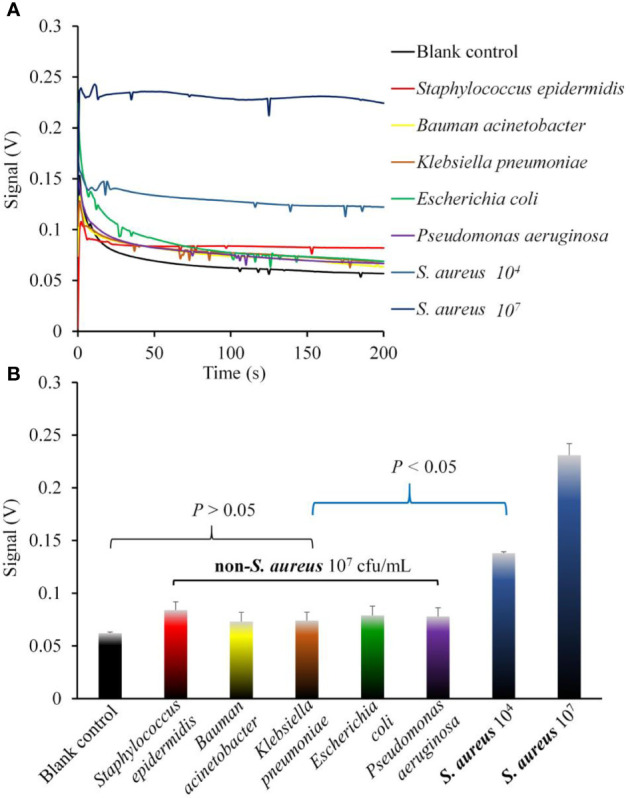
The specificity of the biosensor for the detection of *S. aureus* compared with non-*S. aureus*. **(A)** Signal-time response trace during a complete test cycle (200 s) with the different kinds of pathogenic bacteria. **(B)** The optimal signal detection values of the fluorescence intensity of *S. aureus* and five non-*S. aureus* strains. The statistical analysis suggested that the signal values of all non*-S. aureus* groups were not significantly different from that of the blank control group (*P >*0.05), but all of them were significantly lower than that of both the two *S. aureus* groups (10^4^ and 10^7^ CFU/ml, *P <*0.05). Error bars in **(B)** are based on standard deviations (n = 3).

The fluorescence intensity and the significant difference between *S. aureus* and five non-*S. aureus* strains are shown in **Figure 6**. Signal-time response trace during a complete test cycle (200 s) with the different kinds of pathogenic bacteria are shown in **Figure 6A**. Histogram in **Figure 6B** suggests that the fluorescence intensities of all non-*S. aureus* groups were not significantly different from that of the blank control group (*P >*0.05), but all were significantly lower than those of both *S. aureus* groups (1 × 10^4^ CFU/ml and 1 × 10^5^ CFU/ml, *P <*0.05).

### Detection Results of Clinically Isolated *S. aureus* Strains

**Figure 7:**
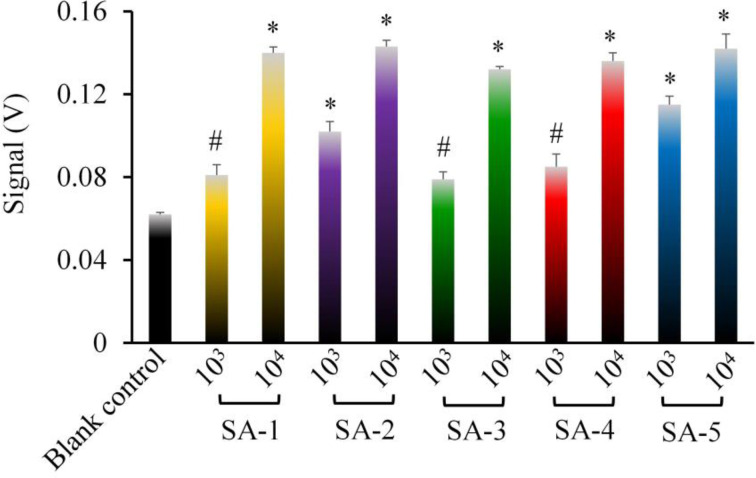
The sensitivity of the biosensor for the detection of the five clinically isolated *S. aureus* strains. The results showed that the sensitivity could reach 10^3^ CFU/ml in the detection of some clinical *S. aureus* strains. * represented *P <*0.05 and ^**#**^ represented *P >*0.05 comparing with the blank control group (PBS). SA-1 to SA-5 represented five *S. aureus* strains isolated from different patients. Error bars are based on standard deviations (n = 3).

The results of the biosensor sensitivities for five clinically isolated *S. aureus* strains are shown in **Figure 7**. The results showed that compared with that of the blank control group (PBS), the fluorescence intensity of the five *S. aureus* groups (1 × 10^4^ CFU/ml) was significantly higher (*P <*0.05). However, although the fluorescence intensity of the five *S. aureus* groups (1 × 10^3^ CFU/ml) was higher than that of the blank control group, there were no significant differences between three (3/5, 60%) of the *S. aureus* strains (SA-2, SA-4, SA-5) and the blank control (*P >*0.05).

## Discussion

*S. aureus* is a common gram-positive pathogenic bacterium with strong pathogenicity, which greatly threatens the health and even life of *S. aureus*-infected patients (Briaud et al., 2020; Gao et al., 2020). Early rapid diagnosis of *S. aureus* is valuable for the timely use of effective antimicrobial therapy to improve prognosis. Previous studies have reported many methods for the rapid detection of *S. aureus*, such as PCR, protein markers, and immunofluorescence, but each method has advantages and disadvantages, as mentioned above, and has not been widely used in clinical practice (Wu et al., 2013; Hirvonen, 2014; Gill et al., 2019).

Biosensors are a multidisciplinary technology that integrates biology with other disciplines, such as physics and chemistry. Since the first oxygen biosensor appeared in 1962, biosensor operation has become simpler, smarter, faster and more accurate due to the advancement of biosensor-related technology. Therefore, there are increasing application studies in clinical and food aetiology detection (Rubab et al., 2018; Cui et al., 2020). In this study, we constructed a new set of quantum dots immunofluorescence biosensors that combined the advantages of biosensors with the high specificity of antigen-antibody immune interactions and the high sensitivity and stability of quantum dots fluorescence. Our research suggested that this biosensor has high specificity and sensitivity and takes only 2 h to produce results, so it can achieve rapid and accurate *S. aureus* detection.

Among all the research results, SEM data showed that the antibody-immobilized optical fiber probe we prepared could specifically capture *S. aureus*, and more *S. aureus* were captured as the concentration of *S. aureus* increased. Additionally, the sensor sensitivity experiment also revealed that the fluorescence intensity detected by the biosensor increased with the increase of the *S. aureus* concentration, and there was a dose-response relationship. Thus, the concentration of *S. aureus* could be roughly estimated by the fluorescence intensity. Additionally, compared with the signal of the *S. aureus*-free control, when the concentration of *S. aureus* was 1 × 10^4^ CFU/ml or greater, the quantum dots fluorescence intensity increased significantly. However, the fluorescence intensity of the 1 × 10^3^ CFU/ml group was not significantly different from that of the control group. This result indicated that the biosensor detection sensitivity for *S. aureus* reached 1 × 10^4^ CFU/ml.

However, we also found that the fluorescence intensity of the 1 × 10^8^ CFU/ml group was not greater than that of the 1 × 10^7^ CFU/ml group. This result was consistent with that of the optical fiber probe capture rate experiment. The capture rate experiment showed that the capture rate of the 1 × 10^8^ CFU/ml group was the lowest although the 1 × 10^8^ CFU/ml group had captured the greatest number of *S. aureus*. The reason we inferred that the quantity of antibody-immobilized to the optical fiber probe was limited, and the number of *S. aureus* in the 1 × 10^8^ CFU/ml group exceeded the maximum capture capacity of the probe. In addition, excessive *S. aureus* may increase non-specific adsorption with poor stability, competitively reducing the capture of specifically bound *S. aureus* with good stability.

*S. aureus* used in the sensitivity experiments was a *S. aureus* standard laboratory strain (ATCC 29213 strain). However, when this biosensor was applied to the detection of five clinically isolated *S. aureus* strains, we found that the fluorescence intensities of two strains (1 × 10^3^ CFU/ml) were significantly higher than that of the blank control (*P <*0.05), while those of the remaining three strains (1 × 10^3^ CFU/ml) were not significantly higher than that of the control (*P >*0.05). The difference in sensitivity of this biosensor between the *S. aureus* standard strain and the clinically isolated *S. aureus* strains may be one of the shortcomings of this biosensor. The specific reason might be related to the complexity of the clinically isolated *S. aureus* strains, such as bacterial activity, gene polymorphism, sources of specimen, and clinical medications. Therefore, we concluded that the detection limit of *S. aureus* reached 1 × 10^4^ CFU/ml or even 1 × 10^3^ CFU/ml in the detection of some clinical strains, and moreover, the fluorescence intensity had a significant positive linear correlation relationship with the logarithm of the *S. aureus* concentration in the range of 10^3^–10^7^ CFU/ml (correlation coefficient R_2_ = 0.9731, *P* = 0.011).

The biosensor specificity experiment showed that the fluorescence intensities of the five most common pathogenic bacteria other than *S. aureus*, such as *E. coli* and *K. pneumoniae*, in nosocomial infections were not significantly different from that of the blank control. However, it was satisfactory that they were all significantly less than those of the two *S. aureus* concentration groups (1 × 10^4^ CFU/ml and 1 × 10^7^ CFU/ml) (*P <*0.05). Therefore, this biosensor could effectively distinguish *S. aureus* (1 × 10^4^ CFU/ml and above) from other common pathogenic bacteria (non-*S. aureus*) in nosocomial infections. The specificity of the biosensor was high enough for *S. aureus* detection.

We believe that the high sensitivity and high specificity of the new biosensor in the detection of *S. aureus* could attribute to the principle used to identify *S. aureus* in our study. This experimental principle was taken into account the dual needs of high sensitivity and high specificity at the same time. For the specificity, we adopt a double-antibody sandwich structure to capture the *S. aureus* and the high specificity could be guaranteed based on the principle of antigen-antibody specific binding. While the various ultra-sensitive biosensors (Li et al., 2018; Andersson et al., 2020; Chen et al., 2020) based on the molecular detection technology of *S. aureus*-related genes may lead to excessively high false positive rate. In particular, even a small amount of contamination in clinical samples can lead to false positives. In addition, for high sensitivity, on the basis of ensuring high specificity, we also adopted two experimental principles to improve the sensitivity of the biosensor. First, we had selected two different kind of antibodies. Among them, the rabbit polyclonal *anti-S. aureus* antibody (ab20920) immobilized on the probe surface was used to capture as many *S. aureus* as possible in order to improve the sensitivity of the biosensor. The other antibody was the mouse monoclonal anti-*S. aureus* antibody (ab37644) and it could ensure the specificity. Second. We used quantum dots (QDs) with obvious optical advantages as the fluorescence signal and combined the signal cascade amplification principle of the biotin–avidin system to promote the sensitivity of the biosensor, instead of other common fluorescence materials (Bhardwaj et al., 2017; Loi and Antelmann, 2020) or just using quantum dots without biotin-avidin system (Shi et al., 2015). Therefore, through the above-mentioned experimental principles, it is ensured that the biosensor has both high sensitivity and high specificity.

In addition, as mentioned in the method about sensitivity experiment, it was speculated that the two *S. aureus* strains (SA-4 and SA-5) might have produced the biofilm in the infection in body, resulting in ineffective antimicrobial treatment, while many previous literatures about the drug resistance related to the *S. aureus* biofilm had been reported (Bui et al., 2017; Grassi et al., 2017; Kavanagh et al., 2018). Bacterial biofilm (BF) is a membrane-like viscous secretion formed by bacteria (Seethalakshmi et al., 2020). Generally, biofilm is produced under microenvironmental conditions that are not suitable for bacterial growth, such as nutritional deficiency, antibacterial drugs, and immune attacks, and the biofilm can protect bacteria from the attack. In the body, the antibacterial drugs cannot reach the minimum inhibitory concentration in the bacterial biofilm, which reduces the bactericidal activity and therapeutic effect of sensitive antibacterial drugs. But there are no good drugs or solvents that can be used to dissolve and liquefy the biofilm in the body to improve the therapeutic effect of antimicrobial drugs. However, *in vitro*, before the bacterial culture and other detection operations of specimens with viscous clinical secretions (such as sputum specimens and skin pus), various digestive juices (such as Sputasol and trypsin digestion solution) can and need to be used to liquefy the specimen, so that the bacterial biofilm and other secretions in the specimen can be digested and decomposed to form a uniform bacterial suspension. Then, the bacterial suspension can be correctly sampled for bacterial culture or other detection operations. While the insufficient liquefaction of the specimens might result in false negative results of bacterial culture due to uneven distribution of pathogenic bacteria in the specimen and incorrect sampling for bacterial culture. Just as the same as the bacterial culture, the clinical biological specimens also need to be fully liquefied to digest and dissolve viscous secretions such as biofilms before the detection procedure of the biosensor in this study. For this reason, the biosensor in this study might not be affected by the interference of biofilms or other secretions and showed good detection results in sensitivity experiment and the clinical application on the detection of *S. aureus*.

However, although the biosensor we constructed have significant advantages, there were also three limitations to be improved. First, our biosensor cannot yet distinguish between methicillin-resistant *S. aureus* (MRSA) and methicillin-sensitive *S. aureus* (MSSA) because antigen-antibody reaction is not specific enough to distinguish between the two, while it is valuable to distinguish MRSA from MSSA because of the need to choose different antibiotics for antibacterial treatment (Guo et al., 2020). However, we believe that this limitation of the biosensor will be resolved in the future with the advancement of related specific antigen–antibody interaction knowledge. Second, we have also applied this biosensor in the detection of more clinical real samples from clinical patients. But in this process, we discovered an important problem as follow. The gold standard for detection of *S. aureus* in real samples is bacterial culture, and in these real samples with positive culture of *S. aureus*, the detection rate of this biosensor was almost 100%. However, the positive rate of bacterial culture is relatively low in clinical real samples, and for some bacterial culture-negative samples (maybe false negative), the detection results of the biosensor were still positive. For this reason, these positive results could not accurately determine whether they are the real *S. aureus* strain infection or just the false positive results. In these cases, the sensitivity and specificity of the biosensor in real samples cannot be accurately judged. However, with the increase in our ongoing application research of clinical real samples, the value of the sensitivity of the biosensor to detect *S. aureus* can get to a much more accurate result, which may not be the current 100%. Similarly, the experimental result of specificity will be more accurate.

In conclusion, we successfully constructed a set of quantum dots immunofluorescence biosensors for *S. aureus* detection. The biosensor possessed high specificity and high sensitivity for the *S. aureus* detection. The concentration of *S. aureus* can reach 1 × 10^4^ CFU/mL or even 1 × 10^3^ CFU/mL and still be detected, and the detection takes only 2 h. Therefore, the sensor can meet the need for rapid and accurate *S. aureus* detection for clinical application.

## Data Availability Statement

The raw data supporting the conclusions of this article will be made available by the authors, without undue reservation.

## Author Contributions

JC and MZ helped to perform the experiment and drafted the manuscript. YingL and ZL completed most of the experiment, especially the detection of strains by the sensor and the imaging of the scanning electron microscope. YuanL and YangL helped the statistical analysis of experimental data and revised the manuscript. YanqL and LY participated in the preparation of the optical fiber probe of the sensor in the experiment. LC and CY designed and provided the fund to support this experiment. All authors contributed to the article and approved the submitted version.

## Funding

This study was supported by the Chinese Military Medical Innovation Project (Grant number 16CXZ041). On behalf of my co-authors, we declare that there was no conflict of interest exited in this study.

## Conflict of Interest

The authors declare that the research was conducted in the absence of any commercial or financial relationships that could be construed as a potential conflict of interest.
