# Supplementary material for: A New Optical Fiber Probe-Based Quantum Dots Immunofluorescence Biosensors in the Detection of Staphylococcus aureus
Source: Front Cell Infect Microbiol. 2021 May 31;11:665241. doi: 10.3389/fcimb.2021.665241 (PMC8203335; doi:10.3389/fcimb.2021.665241)
Supplement: Supplementary file 1 [file DataSheet_1.pdf]

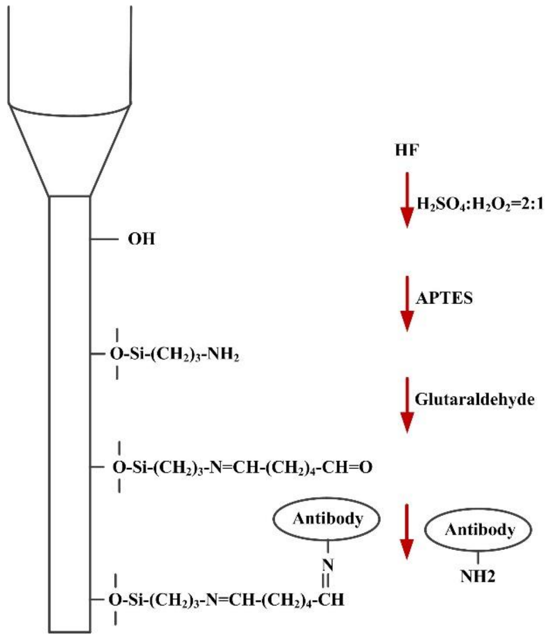

Figure S1. Biological activation of the fiber probe. The cone column combination tapered structure was fabricated with a static tube etching method with 40% hydrofluoric acid (HF). Subsequently, the fiber probe was immersed in a piranha solution (2:1 mixture of concentrated sulfuric acid and 30% hydrogen peroxide) for 30 minutes to remove the residual HF and modify hydroxyl on the surface. Then, the probe were incubated with a 2% (v/v) solution of (3-aminopropyl) triethoxysilane (APTES) in isopropyl alcohol for 1 h on a magnetic stirrer. Afterwards, the probe was baked at 60 for 1 h. After that, a monolayer silane film was covalently bonded on the silica surface of the fiber probe with the amino functional groups. A solution of 1.25% (v/v) glutaraldehyde in PBS buffer (pH=7.4) was used for covalent binding the aldehyde group on the fiber. The probe was immersed in the solution on a magnetic stirrer for 1h, followed by rinsing with PBS buffer. The aldehyde-functionalized fiber probe was then subject to antibody immobilization via amine-aldehyde coupling chemistry. The probe was incubated in rabbit polyclonal anti-S. *aureus* antibody (20 µg/mL in Tris-HCl buffer) at 37 °C overnight in order to allow the aldehyde groups on the probe to covalently bind amino groups of the antibody. After antibody immobilization, the fiber probe was immersed in 1% (w/v) NaBH4 solution for 10 min to reduce the interference of background fluorescence caused by aldehyde group. And the remaining aldehyde groups and non-specific binding cites were blocked by immersing the probe in BSA solution (0.2% (w/v) in PBS) for 1h.
