# Supplementary material for: A New Optical Fiber Probe-Based Quantum Dots Immunofluorescence Biosensors in the Detection of Staphylococcus aureus
Source: Front Cell Infect Microbiol. 2021 May 31;11:665241. doi: 10.3389/fcimb.2021.665241 (PMC8203335; doi:10.3389/fcimb.2021.665241)
Supplement: Supplementary file 2 [file DataSheet_2.pdf]

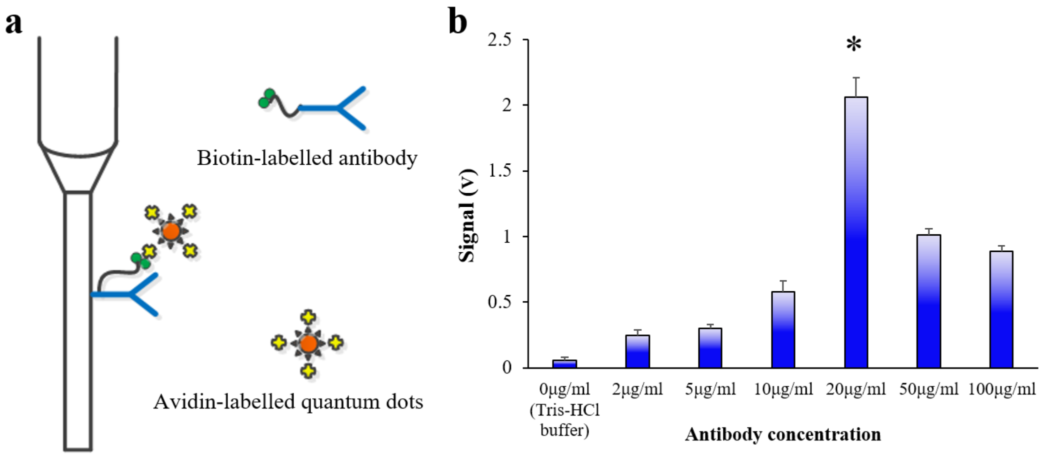

Figure S2. The exploratory research on the optimal concentration of the rabbit polyclonal anti-*S. aureus* antibody (ab20920) used to immobilize onto the probe surface. As the same as the antibody (ab20920) immobilized on the probe surface, the biotin-labelled goat anti-mouse IgG antibody applied in this study was also a polyclonal antibody and it was labelled with biotin. Therefore, it was used as an analog to explore the optimal concentration of the antibody (ab20920) used to immobilize onto the probe surface. (a) Schematic diagram of experimental principle: the biotin-labelled goat anti-mouse IgG antibody was immobilized onto the surface of the probe, and the avidin-labelled quantum dots solution (QDs-605, 1:100 dilution) was bound with the biotin-labelled goat anti-mouse IgG antibody via avidin-biotin conjugation. The relative amount of antibody loaded on the probe is indirectly evaluated by the magnitude of the fluorescent signal emitted from the QDs. (b) The biotin-labelled goat anti-mouse IgG antibody was diluted with Tris-HCl buffer to different antibody concentrations as shown in the figure and the Tris-HCl buffer was as the control group. And the results of the optimal detection values of the fluorescence signals of the various groups had clearly confirmed that the 20ug/ml was the optimal concentration of the antibody used to immobilize onto the probe surface. * represented $P<0.05$ comparing with other groups. Error bars in (b) are based on standard deviations (n=3).
