# Supplementary material for: A New Optical Fiber Probe-Based Quantum Dots Immunofluorescence Biosensors in the Detection of Staphylococcus aureus
Source: Front Cell Infect Microbiol. 2021 May 31;11:665241. doi: 10.3389/fcimb.2021.665241 (PMC8203335; doi:10.3389/fcimb.2021.665241)
Supplement: Supplementary file 3 [file Table_1.docx]

**Table S1.** The EDX analysis of the SEM images of *S. aureus* and blank area of the fiber probe

| *S. aureus* | | |  | Blank area | | |
| --- | --- | --- | --- | --- | --- | --- |
| Element | Weight % | Atomic % |  | **Element** | Weight % | Atomic % |
| C K | 29.82 | 43.72 |  | **C K** | 8.67 | 14.26 |
| O K | 25.96 | 28.57 |  | **O K** | 40.63 | 50.14 |
| Na K | 1.00 | 0.76 |  | **Na K** | 0 | 0 |
| Si K | 41.99 | 26.33 |  | **Si K** | 50.36 | 35.41 |
| Cl K | 1.23 | 0.61 |  | **Cl K** | 0.34 | 0.19 |
